# Humic Acid Increases Amyloid β-Induced Cytotoxicity by Induction of ER Stress in Human SK-N-MC Neuronal Cells

**DOI:** 10.3390/ijms160510426

**Published:** 2015-05-07

**Authors:** Hsin-Hua Li, Fung-Jou Lu, Hui-Chih Hung, Guang-Yaw Liu, Te-Jen Lai, Chih-Li Lin

**Affiliations:** 1 Institute of Medicine, Chung Shan Medical University, Taichung 40201, Taiwan; E-Mails: vivid529@hotmail.com (H.-H.L.); fjlu@csmu.edu.tw (F.-J.L.); 2 Department of Life Sciences and Institute of Genomics and Bioinformatics, National Chung Hsing University, Taichung 40227, Taiwan; E-Mail: hchung@dragon.nchu.edu.tw; 3 Institute of Microbiology & Immunology, Chung Shan Medical University, Taichung 40201, Taiwan; E-Mail: liugy@csmu.edu.tw; 4 Department of Psychiatry, Chung Shan Medical University Hospital, Taichung 40201, Taiwan; 5 Department of Medical Research, Chung Shan Medical University Hospital, Taichung 40201, Taiwan

**Keywords:** amyloid β, endoplasmic reticulum stress, humic acid, PPARγ coactivator 1α, sirtuin 1

## Abstract

Humic acid (HA) is a possible etiological factor associated with for several vascular diseases. It is known that vascular risk factors can directly increase the susceptibility to Alzheimer’s disease (AD), which is a neurodegenerative disorder due to accumulation of amyloid β (Aβ) peptide in the brain. However, the role that HA contributes to Aβ-induced cytotoxicity has not been demonstrated. In the present study, we demonstrate that HA exhibits a synergistic effect enhancing Aβ-induced cytotoxicity in cultured human SK-N-MC neuronal cells. Furthermore, this deterioration was mediated through the activation of endoplasmic reticulum (ER) stress by stimulating PERK and eIF2α phosphorylation. We also observed HA and Aβ-induced cytotoxicity is associated with mitochondrial dysfunction caused by down-regulation of the Sirt1/PGC1α pathway, while in contrast, treating the cells with the ER stress inhibitor Salubrinal, or over-expression of Sirt1 significantly reduced loss of cell viability by HA and Aβ. Our findings suggest a new mechanism by which HA can deteriorate Aβ-induced cytotoxicity through modulation of ER stress, which may provide significant insights into the pathogenesis of AD co-occurring with vascular injury.

## 1. Introduction

According to the most widely accepted humification theories, humic acids (HA) are recalcitrant and refractory high molecular weight heteropolymers occurring in peat, soils, sediments and waters [1,2], and are characterized as possible etiological factors for several vascular diseases such as Blackfoot disease (BFD) [3]. BFD is a unique peripheral vascular disease (PVD) endemic confined to the southwestern coast of Taiwan [4]. These diseases result in circulation disorders that affect blood vessels and cause endothelial dysfunction [5]. In addition, evidence indicates that PVD is also a risk factor for cerebrovascular disease (CVD) [6]. Because CVD tends to co-occur in cognitive function, it is reasonable to propose that PVD patients might also suffer impairment in cognitive function due to concomitant CVD [7]. In fact, PVD patients appear to be particularly at risk for vascular-related cognitive deficits [8]. Furthermore, vascular pathologies in brains are highly prevalent in the elderly and closely related to cognitive impairment in later life [9]. These observations suggest that consuming excessive amounts of HA may plausibly have adverse effects on vascular cognitive impairment caused by damaged brain blood vessels, such as PVD affects peripheral vascular system. However, little is known about the underlying pathophysiological mechanisms of HA that might contribute to the progression of cognitive impairments.

A number of studies suggest a link between vascular risk factors and amyloid β (Aβ) deposits, one of major pathogenic events for Alzheimer’s disease (AD) [10]. Although a current concept that the presence of Aβ deposits is the major contributor to dementia, vascular brain injury shows a greater correlation with impaired cognitive function than Aβ concentrations [11]. It is known that vascular brain injury is accompanied by evidence of Aβ deposition, and are both associated with cognitive decline in aging [12]. Moreover, studies in both AD brains and animal models also demonstrated a direct and positive link between vascular and Aβ pathology [13,14]. These observations explain why clinical trials for anti-Aβ treatment may fail if patients are demented by vascular problems [15], and indicate the neurotoxic effects of Aβ may be enhanced by a subsequent vascular deleterious event such as HA. Therefore, it is conceivable that exposure to large quantities of HA may have adversely affected the Aβ-induced neurotoxicity which is conducive to AD pathogenesis. However, to the best of our knowledge, no link has been reported between HA and Aβ-induced neurotoxicity.

Increased oxidative stress has been suggested to play a critical role in patients with PVD [16]. Oxidative stress is a state of excessive reactive oxidative species (ROS) generation, which can result in cellular injury and death. Increased levels of ROS cause mitochondrial dysfunction and trigger several cell death pathways in the brain [17]. Previous studies show that excessive endoplasmic reticulum (ER) stress may initiate cell death cascades that are mediated, in part, through mitochondrial dysfunction [18]. During ER stress, the protein kinase RNA-like ER-localized kinase (PERK) pathway is activated through phosphorylation of PERK and eukaryotic initiation factor 2α (eIF2α), and results in stimulating caspase-4 dependent apoptosis [19]. Furthermore, suppression of Aβ-induced ER stress upregulates the level of peroxisome proliferator-activated receptor γ coactivator 1α (PGC1α) [20], which is known as a pro-survival transcriptional coactivator that plays a central role in mitochondrial biogenesis and function. This suggests ER stress may enhance the cytotoxicity of Aβ and make neuronal cells more vulnerable to stress-induced neurodegeneration. In our previous studies, HA damaged pancreatic islet cells through increased oxidative stress and depleted several antioxidant enzymes [21,22]. Moreover, HA-induced apoptosis can also be mediated through the ER stress, caspase 4 and mitochondria-dependent pathways in macrophages, indicating that HA exposure may also potentiate oxidative stress and ER stress that are associated with neurodegeneration. However, HA-induced toxicity has not been well demonstrated in neurodegenerative diseases such as AD. In this work, we therefore investigated the effects of HA on Aβ-induced cell death in human SK-N-MC neuronal cells. The effective combination and the molecular target pathways, in particular for ER stress signals and oxidative stress-associated mitochondrial dysfunction, were also investigated.

## 2. Results

### 2.1. Effects of Humic Acid (HA) on Aβ-Induced Cytotoxicity

**Figure 1:**
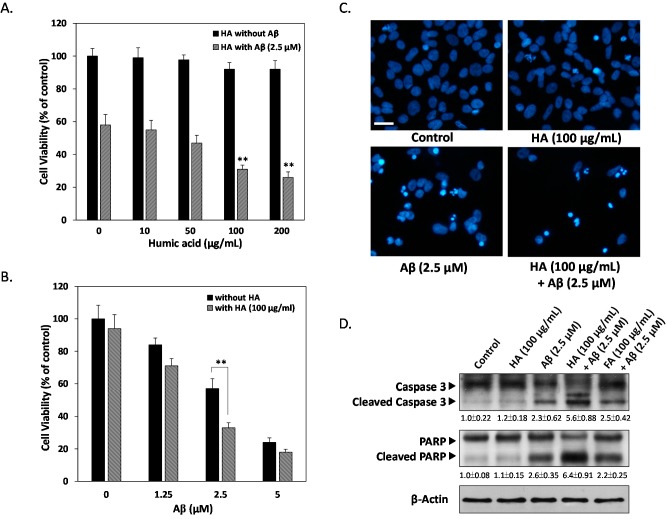
Humic acid (HA) enhances exogenous Aβ-induced apoptosis in human SK-N-MC cells. (**A**) Cells were treated with either 0, 10, 50, 100 or 200 μg/mL HA, and co-treated with or without 2.5 μM Aβ for 24 h followed by 3-(4,5-dimethylthiazol-2-yl)-2,5-diphenyltetrazolium bromide (MTT) assays; (**B**) Cells were treated with either 0, 1.25, 2.5 or 5 μM Aβ with or without 100 μg/mL HA for 24 h followed by MTT assays. Co-treatment of the cells with HA further increased Aβ-induced cell death compared to the Aβ alone groups; (**C**) HA markedly increased Aβ-induced nucleus fragmentation. Apoptosis was determined by fragmented morphology in nuclei for DAPI fluorescence; (**D**) Aβ-induced cell death was determined by western blotting of cleaved caspase 3 and PARP levels, showing a synergistically enhanced apoptosis after HA concomitant treatments. HA, humic acid. FA, fulvic acid. All results are shown from three independent experiments, and values are presented as mean ± SEM. Significant differences was determined by using the multiple comparisons of Dunnett’s *post-hoc* test for ** *p* < 0.01. Scale bar represents 50 μm.

**Figure 2:**
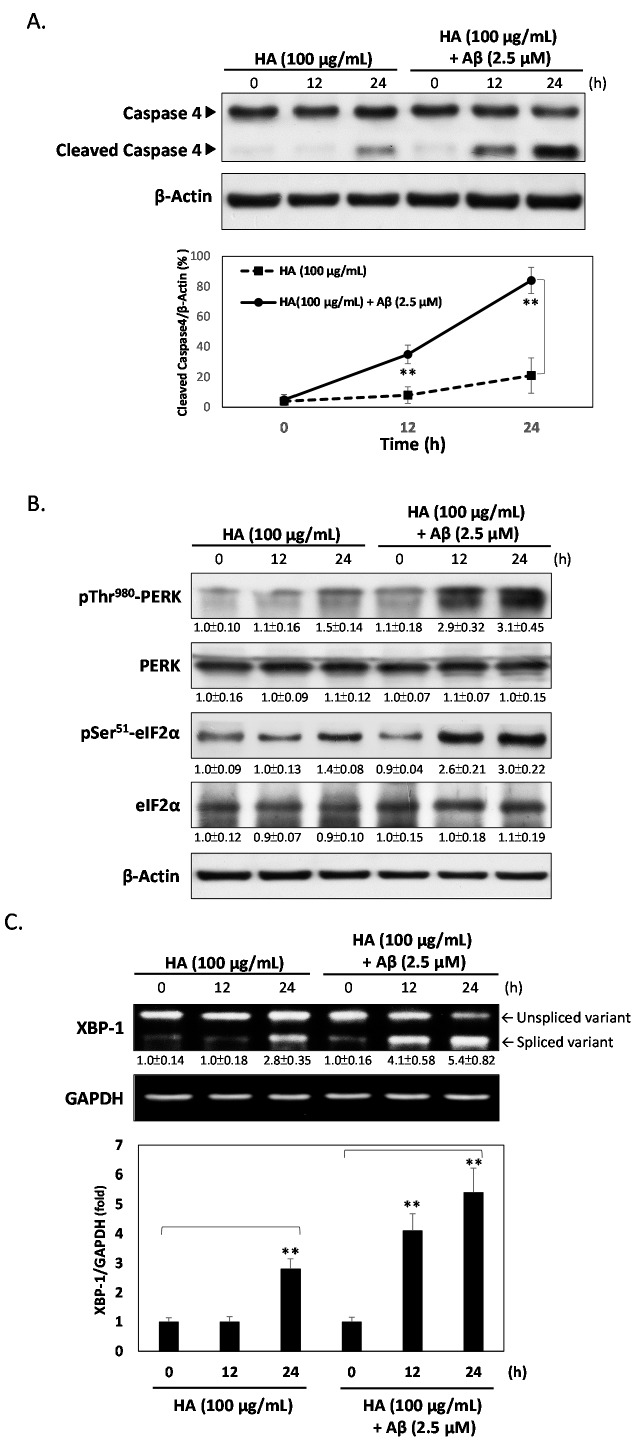

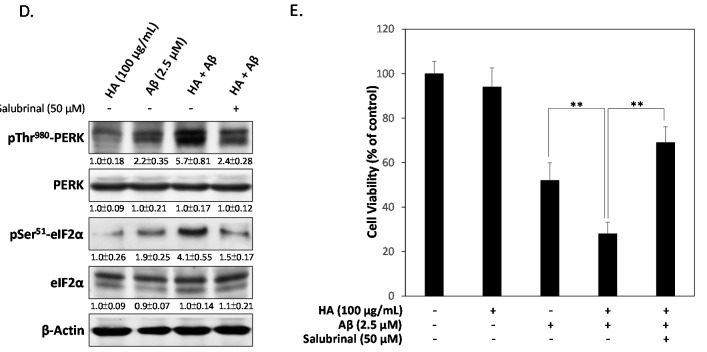
HA increases Aβ-induced ER stress. SK-N-MC cells were treated with 100 μg/mL HA with or without 2.5 μM Aβ for 0 to 24 h. (**A**) Immunoblotting of caspase 4 showed HA causes cleavage of caspase 4, and that co-treatment with Aβ increases this cleavage in a time-dependent manner. The lower panel displays quantitative analyses showing cleaved caspase 4/β-actin ratios; (**B**) Western blots showed the activation of the ER stress by monitoring the phosphorylation of PERK and eIF2α in cells treated with HA alone, or in combination with Aβ for 0 to 24 h; (**C**) RT-PCR analysis showed the splicing of XBP-1 mRNA for the indicated time in cells treated with HA alone, or in combination with Aβ for 0, 12 or 24 h; (**D**) Salubrinal, an ER stress inhibitor blocked HA and Aβ-mediated ER stress-induced PERK and eIF2α phosphorylation at 24 h; (**E**) Cell viabilities were significantly increased in Salubrinal-treated cells compared to HA and Aβ co-treated cells for 24 h. All results are shown from three independent experiments, and values are presented as mean ± SEM. Significant differences was determined by using the multiple comparisons of Dunnett’s *post-hoc* test for ** *p* < 0.01.

To understand the effects of HA on neuronal cells, human neuronal SK-N-MC cells were exposed to various HA concentrations ranging from 10 to 200 μg/mL for 24 h. As shown in Figure 1A, no significant decrease on cell viability was observed with HA treatments up to 200 μg/mL, indicating that no toxic effects were present when HA was administered alone. However, cells incubated with 2.5 µM of exogenous Aβ for 24 h markedly underwent a ~50% decrease of 3-(4,5-dimethylthiazol-2-yl)-2,5-diphenyltetrazolium bromide (MTT) reduction, and this effect could be enhanced by the concomitant presence of 100 μg/mL of HA (Figure 1B). This indicates HA significantly enhances Aβ-induced cell viability loss from 57% ± 6.1% to 33% ± 3.1%. To precisely determine which mode of cell death is induced by Aβ and HA, we examined nuclei fragmentation by DAPI staining. Figure 1C showed disrupted nucleus margin, indicating Aβ-caused apoptotic events were enhanced by co-treatment of HA. Further studies with apoptotic markers caspase 3 and PARP confirmed that Aβ-induced activation of apoptosis was synergistically elevated in HA-treated cells but not those treated with fulvic acid (FA), one of the major monomers that make up humic substances (Figure 2D). Taken together, these findings confirm the specific role of HA in enhanced Aβ-induced apoptosis in SK-N-MC neuronal cells.

### 2.2. HA Increases Aβ-Induced Endoplasmic Reticulum (ER) Stress

It has been shown that HA-induced apoptosis may be mediated through the ER stress pathway [23]. To investigate whether HA regulates the Aβ-induced cytotoxicity via stimulating ER stress, we performed western blotting of caspase 4, a critical marker of ER stress. As shown in Figure 2A, HA caused a slight increase in the expression of cleaved caspase 4 after 24 h treatment. However, co-treatment of Aβ markedly increased cleaved caspase 4 expression. To confirm the role of this pathway, the phosphorylation levels of downstream typical markers of ER stress such as PERK, eIF2α, and XBP-1 were also evaluated. As shown in Figure 2B, co-treatment with HA and Aβ for 24 h significantly increased the phosphorylation of PERK on threonine 980 and eIF2α on serine 51, respectively. Similarly, the XBP1 mRNA splicing revealed that the splicing activity was enriched in co-treatment with HA and Aβ (Figure 2C). To further clarify mechanisms underlying ER stress-mediated apoptosis, the eIF2α inhibitor Salubrinal was conducted to interfere with HA and Aβ-mediated ER stress responses. As shown in Figure 2D, Salubrinal inhibited the levels of p-Thr980 PERK and p-Ser51 eIF2α in HA and Aβ co-treated cells, indicating that HA enhances Aβ-induced ER stress. Additionally, Salubrinal also increased cell viability in HA and Aβ co-treated cells, suggesting that the cytotoxic effects of HA and Aβ indeed function through activation of ER stress (Figure 2E).

### 2.3. HA Exacerbates Aβ-Induced Mitochondria Dysfunction and ROS Accumulation

**Figure 3:**
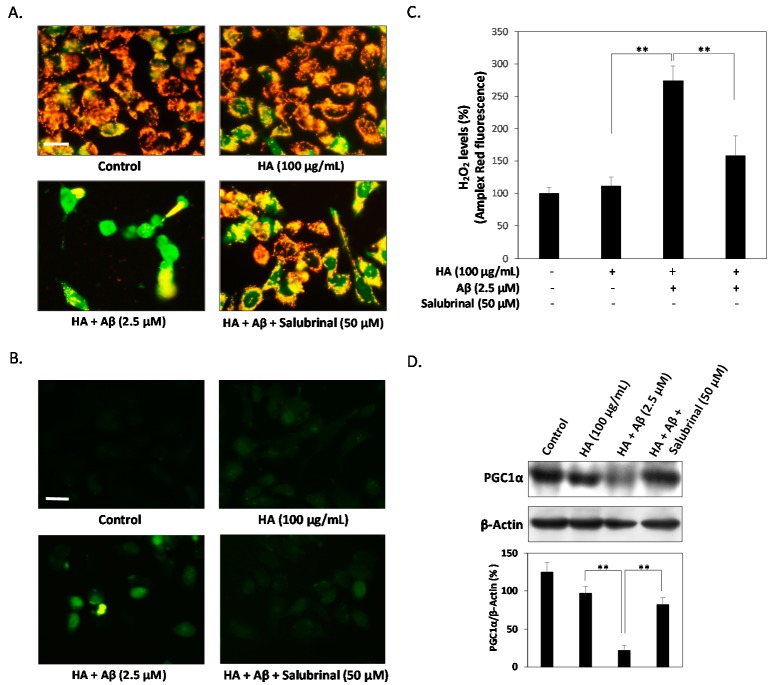

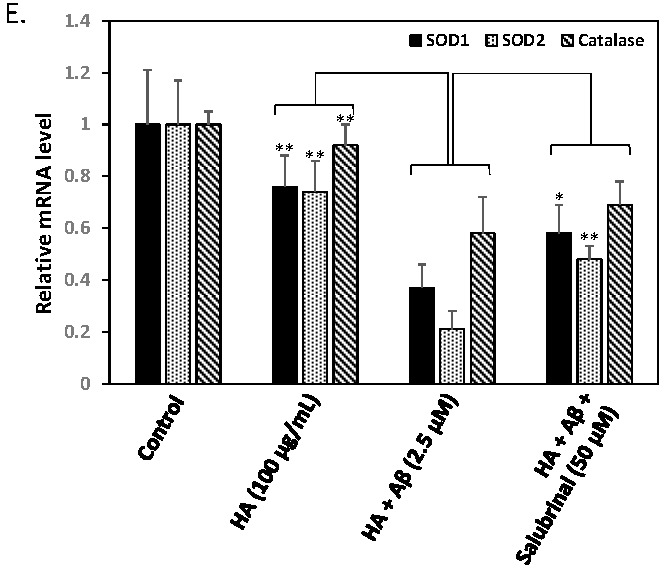
HA increases Aβ-induced mitochondria dysfunction and H_2_O_2_ production. (**A**) The mitochondrial membrane potential stained with JC-1 dye was observed by fluorescent microscopy. After co-treatment of HA and Aβ, SK-N-MC cells showed more intense green fluorescence than non-treated and Salubrinal-treated cells; (**B**) Intracellular oxidative bursts were determined by dichlorofluorescin diacetate (DCFH-DA) staining. Cells were treated with the indicated compounds for 24 h. After treatment, cells were stained by DCFH-DA and visualized by using fluorescence microscopy; (**C**) Measurements of H_2_O_2_ production from SK-N-MC cells by Amplex red fluorometric method. Co-treatment of HA and Aβ significantly induced H_2_O_2_ production parallel to decreased mitochondrial membrane potential; (**D**) Western blotting assays showed that co-treatment of HA and Aβ inhibits PGC1α expression, whereas the addition of Salubrinal significantly returns this inhibition; (**E**) The mRNA levels of SOD1, SOD2 and catalase were detected by real time PCR, showing that HA and Aβ-treated SK-N-MC cells express significantly less mRNA than non-treated and Salubrinal-treated cells. All results are shown from three independent experiments, and values are presented as mean ± SEM. Significant differences were determined by using the multiple comparisons of Dunnett’s *post-hoc* test for * *p* < 0.05 and ** *p* < 0.01. Scale bar represents 50 μm.

Since it is recognized that Aβ induces ROS accumulation to decrease mitochondrial membrane potential and increase oxidative damage, the role of HA in deteriorating Aβ-induced ROS accumulation was also investigated. As shown in Figure 3A, only a slight deficiency of mitochondrial membrane depolarization was detected in HA-treated cells but a significant deficiency was observed in Aβ co-treated groups, as determined by the concurrent loss of cytoplasmic red JC-1-aggregate fluorescence and increase of diffused green fluorescence. In contrast, this deficiency was markedly diminished by the addition of Salubrinal (50 μM). Previous studies also show strong evidence that Aβ-induced ROS accumulation and mitochondrial dysfunction are both potential pathogenic markers in AD. To determine whether HA increases Aβ-induced oxidative stress, we measured intracellular oxidative levels by 2',7'-dichlorofluorescin diacetate (DCFH-DA) and Amplex red fluorometric methods. As shown in Figure 3B, our results demonstrated that co-treatment of HA and Aβ markedly increased intracellular oxidative bursts. Figure 3C further shows that co-treatment of HA and Aβ significantly increased the levels of H_2_O_2_ present in SK-N-MC cells. However, this increase in oxidative burst and H_2_O_2_ were diminished in Salubrinal-treated cells, indicating that HA and Aβ induce intracellular superoxide radical anion accumulation and mitochondria dysfunction via stimulating the ER stress signaling pathway.

To prevent the ROS-associated mitochondrial damage and dysfunction, proliferator-activated receptor gamma coactivator 1 α (PGC1α) has been reported as a positive regulator of mitochondrial function and ROS scavenging enzymes [24]. Therefore, we next investigated whether HA mediates PGC1α expression to induce the damages of intracellular superoxide radical anion accumulation. As shown Figure 3C, HA caused a slight decrease in the expression of PGC1α at 24 h, and this inhibition was markedly increased by co-treatment of Aβ. However, the addition of Salubrinal significantly returned the PGC1α expression. Moreover, qPCR analysis showed that co-treatment of HA and Aβ significantly inhibited the SOD1, SOD2 and catalase mRNA expression in SK-N-MC cells, and these inhibitions were also returned by the addition of Salubrinal at 24 h (Figure 3D). Taken together, our findings clearly demonstrated that HA exacerbates Aβ-induced mitochondria dysfunction and ROS accumulation via stimulating ER stress.

### 2.4. HA and Aβ-Induced ER Stress Disturbs Sirt1/PGC1α Signaling

**Figure 4:**
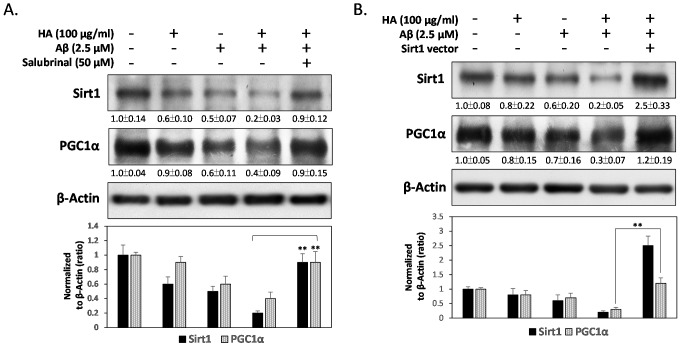

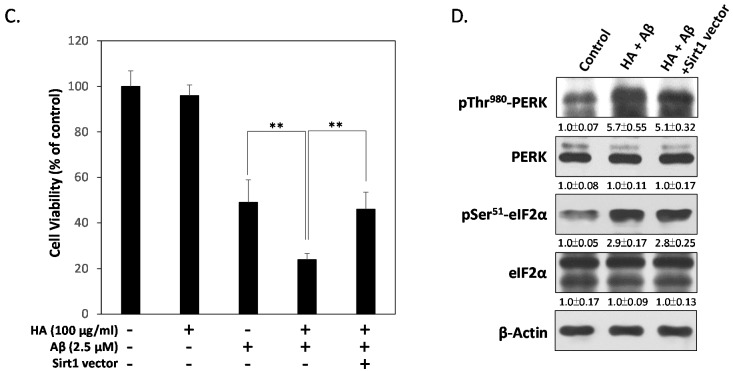
Sirt1 protects neuronal cells from HA and Aβ-induced PGC1α suppression and cytotoxicity. (**A**) Western blotting assays showed the co-treatment of HA and Aβ inhibits expressions of Sirt1 and PGC1α. However, the addition of Salubrinal (50 μM) significantly blocked these inhibitions; (**B**) Sirt1 over-expression significantly restores HA and Aβ-inhibited PGC1α levels, (**C**) and contributes to a better survival rate in HA and Aβ co-treated cells; (**D**) Sirt1 over-expression failed to inhibit the levels of p-Thr980 PERK and p-Ser51 eIF2α in HA and Aβ co-treated cells, indicating that ER stress occurs upstream of Sirt1 down-regulation. All results are shown from three independent experiments, and values are presented as mean ± SEM. Significant differences were determined by using multiple comparisons of Dunnett’s *post-hoc* test for ** *p* < 0.01.

In view of all the above evidence, we concluded that the co-treatment of HA and Aβ mediates ER stress to suppress PGC1α-associated mitochondrial function and ROS scavenging enzymes. To investigate whether HA and Aβ interferes with the expression of Sirt1, an upstream regulator of PGC1α, western blotting was performed. As shown in Figure 4A, the levels of both Sirt1 and PGC1α were significantly decreased by co-treatment with HA and Aβ. However, the addition of Salubrinal significantly blocked these inhibitions, indicating that HA and Aβ-induced ER stress represses the Sirt1/PGC1α signaling pathway. To further investigate whether Sirt1 plays a role in protecting neuronal cells from HA and Aβ-induced cytotoxicity, we transiently transfected SK-N-MC cells with a pcDNA3.1 vector overexpressing Sirt1. As shown in Figure 4B, Sirt1 over-expression relieved HA and Aβ-induced down-regulation of PGC1α significantly. Moreover, Sirt1 over-expression also inhibited the cytotoxicity in HA and Aβ co-treated cells, indicating a protective role of Sirt1 in preserving PGC1α to prevent HA and Aβ-induced cytotoxicity (Figure 4C). However, Figure 4D further showed Sirt1 over-expression fails to attenuate HA and Aβ-induced phosphorylation of PERK and eIF2α, suggesting that HA and Aβ-induced ER stress may result in the down-regulation of Sirt1.

## 3. Discussion

Aβ deposits and brain vascular injury are the two most common pathologic changes in the aging brain. Although HA is a potential toxin leading to vascular injury observed for patients [4], the molecular mechanism underlying HA involving Aβ-induced neurotoxicity has not been elucidated. Our study demonstrates for the first time that HA can exhibit a synergistic effect enhancing Aβ-induced neurotoxicity in human neuronal cells. These deteriorative effects of HA are mediated through the activation of ER stress signaling by stimulating PERK and eIF2α phosphorylation. Moreover, this ER stress activation is associated with a decrease in the intracellular levels of Sirt1, which subsequently represses PGC1α and leads to mitochondrial dysfunctions including the loss of mitochondrial membrane potential and accumulation of ROS associated with apoptosis. Therefore, the presented data provide important evidence that HA-induced ER stress is associated with Aβ-induced neuronal death, and can partially explain how vascular risk factors such as HA are involved in the pathogenesis of AD.

The exact sequence of events whereby Aβ causes neurodegeneration in AD is not fully known. However, there is significant evidence that links mitochondrial dysfunction and oxidative damage to AD development and progress [25]. Particularly, mitochondrial damage correlates with increased intracellular production of oxidants, which is implicated as the primary cause of vascular lesion mediated AD [26]. As a result, maintaining a healthy mitochondrial function within the neuronal cells eventually plays a role in delaying, slowing down, or even preventing AD. To this, several previous studies have identified PGC1α is the master regulator of mitochondria biogenesis and function [27]. It is well established that PGC1α is intimately linked to both increases in mitochondrial functions and the detoxification of ROS [28]. Thus, it is not surprising that dysregulated PGC1α has been implicated in several neurodegenerative disorders such as AD [28,29]. In fact, it has been reported that the expression of PGC1α is significantly reduced in AD patients and Tg2576 transgenic mice [30]. Studies of the impact of PGC1α on neurodegeneration have also highlighted a potential role for PGC1α in neuroprotection [31]. However, few studies have dealt with the effect of HA on Aβ potentiated mitochondrial dysfunction and oxidative stress. To further understand Aβ-associated neurotoxic mechanisms, our results demonstrate that HA could elevate ER stress with concomitant changes in PGC1α protein content that impairs mitochondria function. In fact, similar results were found in liver cells where hepatic ER stress activation corresponded to the inhibition of PGC1α [32]. In sum, our results indicate that the loss of PGC1α expression increases mitochondrial oxidative stress through exposure to HA which may contribute to Aβ-induced neurotoxicity.

This study also validates that ER-stress-decreased Sirt1 may enhance Aβ-induced neurotoxicity via suppressing PGC1α expression. Mammalian Sirt1 is a protein deacetylase related to an increase in mitochondrial function and antioxidant protection [33]. Recent evidence suggests that Sirt1 interacts with and deacetylates PGC1α at multiple lysine sites, which increases PGC1α activity and leads to the induction of downstream genes [34]. Therefore, Sirt1 is the key effector against Aβ neurotoxicity by activation of PGC1α-mediated mitochondrial activity. Moreover, Sirt1 promotes cell survival through stimulating endogenous antioxidant defense system. For example, Sirt1 and PGC1α appear to co-regulate expression of genes encoding enzymes involved in the ROS defense system including SODs and catalase, leading to alleviated mitochondrial oxidative stress in neuronal cells [35]. Furthermore, our data also demonstrates that Sirt1 over-expression inhibits Aβ-induced cell apoptosis, through HA and Aβ-induced ER stress following PGC1α suppression. As Sirt1/PGC1α signaling has been shown to enhance neuron survival in AD, understanding the mechanism of HA and Aβ-induced ER stress activation may provide novel insights in preventing Aβ-induced neurotoxicity.

HA are heterogeneous organic compounds occurring in soils, sediments and waters with geographical variability [36,37], and have the capacity to interact with several metal ions, which may cause damage to various tissues including brains [38]. Therefore, complex mixtures can be heterogeneous, making results difficult to reproduce and interpret. To consider a precise toxicity evaluation of HA, we use synthetic HA rather than natural purified HA as our study materials. In this study, we demonstrated the effects of HA on Aβ-induced cell death in SK-N-MC neuroblastoma cells. The ideal source of neuronal cells in our experiments is hippocampal neurons from human brains. However, it has long been known that mature and differentiated human neurons do not divide. To circumvent these problems, animal primary neuron cultures are widely used in Aβ-induced cytotoxicity studies. However, cultured primary neurons are difficult to transfect, and the maintenance of stable expression of target genes is challenging because they are post-mitotic and very sensitive to culture conditions. Besides, AD develops aging-related loss of function in older but not embryonic and neonatal primary neuronal cells. As a result, we used SK-N-MC cells as our experimental model based on previous studies [39]. However, further *in vivo* studies are needed to determine whether HA can penetrate into the brain and affect the neurotoxicity of Aβ, and whether these mechanisms are involved in ER stress activation. In conclusion, our findings clearly demonstrate that HA-enhanced ER stress significantly enhances Aβ-induced cytotoxicity. Particularly, alterations of Sirt1/PGC1α expression may serve as a diagnostic maker as well as a therapeutic target for AD co-occurring with brain vascular injury.

## 4. Experimental Section

### 4.1. Materials

Chemicals such as 3-(4,5-dimethylthiazol-2-yl)-2,5-diphenyltetrazolium bromide (MTT), 4',6-diamidino-2-phenylindole (DAPI), dichlorodihydrofluorescin diacetate (DCFH-DA), JC-1, and fulvic acid were purchased from Sigma (Munchen, Germany). Amyloid β (Aβ) 1-42 was acquired from AnaSpec Inc. (San Jose, CA, USA). Salubrinal was obtained from Calbiochem (Darmstadt, Germany). We purchased antibodies against PERK, pPERK, eIF2α, peIF2α, PGC1α and poly (ADP-ribose) polymerase (PARP) from Santa Cruz Biotechnology (Santa Cruz, CA, USA), and those against caspase 3 and caspase 4 from Millipore (Bedford, MA, USA), β-actin antibody from Novus Biologicals (Littleton, CO, USA), and Sirt1 antibody from GeneTex (Irvine, CA, USA).

### 4.2. Preparation of Synthetic Humic Acid (HA)

It has been known that natural HA are complex and heterogeneous mixtures with geographical variability. To eliminate impurities including heavy metal ions, HA was synthesized from monomeric protocatechuic acid according to our previously published procedure [23]. Briefly, 1 g of protocatechuic in 100 mL of distilled water was oxidized with sodium periodate for 24 h in a water bath at 50 °C with shaking for oxidative polymerization. After centrifugation, the supernatant was acidified to pH 1.0 by HCl. The acidified solution was again centrifuged, and its precipitate was treated with 0.1 N NaOH to solubilize HA, which was further purified by absorption chromatography with XAD-7 resin and fractionated by Sephadex G-25 chromatography, as detailed by Hseu *et al.* [40].Then the HA solution was ultrafiltered through a Molecular/Por membrane to exclude particles below 500 Da *M*w. The resultant HA was collected for using in this study.

### 4.3. Cell Culture and Viability Assay

Human neuronal SK-N-MC cells were obtained from the American Type Culture Collection (Bethesda, MD, USA). Human full-length human Sirt1 was obtained from Addgene (Cambridge, MA, USA) and cloned into a pcDNA3.1 expression vector. The Sirt1 plasmid was transiently transfected for over-expression into cells using lipofectamine 2000 reagent (Invitrogen, Carlsbad, CA, USA). Cells were maintained in Minimal Eagle’s medium (MEM; Gibco, Carlsbad, CA, USA), supplemented with 10% fetal calf serum, 100 units/mL penicillin, 100 µg/mL streptomycin, and 2 mM l-glutamine at 37 °C, 5% CO_2_. Solutions of Aβ peptides were prepared by dissolution in 60 mM NaOH, and diluted with 10 mM sodium phosphate (pH 7.4) as a stock reagent. For cell viability assays, cells were seeded in 96-welled plates at a density of 1 × 10^4^ cells/well overnight. Cells were treated to HA concentrations ranging from 10 to 200 μg/mL with or without 2.5 μM of Aβ_1–42_ as indicated. After 24 h, the tetrazolium salt MTT was added to the medium following the manufacturer’s instructions. Only viable cells could metabolize MTT into a purple formazan product, of which the color optical density (OD) wavelength value was further quantified by a Bio-Rad spectrophotometer at 550 nm. Cell viability was determined by the percentage of OD value from treated cells divided by OD value from controls.

### 4.4. Nuclei Morphology by 4',6-Diamidino-2-phenylindole (DAPI) Staining

Changes in cell nucleus morphology, in particular characteristics of apoptosis, were examined in cells grown on coverslips, using a fluorescence microscope. The cells were fixed in 4% paraformaldehyde after 24 h of treatment with the indicated compounds, permeabilized in ice-cold methanol, and incubated for 15 min at room temperature with 1 ng/mL of 4',6-diamidino-2-phenylindole (DAPI) stain under a fluorescence microscope (DP80/BX53, Olympus, Tokyo, Japan).

### 4.5. Western Blot Analysis

Cells were harvested and homogenized in a protein extraction lysis buffer (50 mM Tris-HCl, pH 8.0; 5 mM EDTA; 150 mM NaCl; 0.5% Nonidet P-40; 0.5 mM phenylmethylsulfonyl fluoride; and 0.5 mM dithiothreitol) and centrifuged at 12,000× *g* for 30 min at 4 °C. The supernatants were used as cell extracts for immunoblotting analysis. SDS-solubilized samples were loaded onto SDS-polyacrylamide gels. Equal protein amounts of total cell lysates were resolved by 10% SDS-PAGE, transferred onto polyvinylidene difluoride membranes (Millipore, Bedford, MA, USA), and then probed with a primary antibody followed by secondary antibody conjugated with horseradish peroxidase. The immunocomplexes were visualized with enhanced chemiluminescence kits (Millipore).

### 4.6. Reverse Transcription Polymerase Chain Reaction (RT-PCR) Analysis of XBP1 mRNA Splicing

Total RNA was isolated from SK-N-MC cells by using an RNeasy mini kit (Qiagen, Crawley, UK) immediately after treatments. To amplify XBP-1 mRNA, PCR was carried out for 30 cycles using the following profile: 94 °C for 30 s; 58 °C for 30 s; and 72 °C for 1 min. In the final cycle, PCR products were incubated at 72 °C for an additional 10 min with XBP-1 forward and reverse primers and TaqDNA polymerase (Invitrogen). Forward and reverse primer sequences were 5'-CTGGAACAGCAAGTGGTAGA-3' and 5'-CTGGGTCCTTCTGGGTAGAC-3', respectively. As a previous report indicated β-actin was an unsuitable internal control for RT-PCR, we used GAPDH as our internal standard in this experiment [41].

### 4.7. Analysis of Mitochondrial Membrane Potential

Mitochondrial function was investigated using a vital mitochondrial cationic dye JC-1, which exhibits potential-dependent accumulation in mitochondria. Cells were treated with 1 μM of JC-1 in fresh medium and incubated at 37 °C for 30 min. The staining medium was then carefully discarded and cells were washed twice with PBS. Cell morphology was then observed and photographed using an inverted fluorescence microscope (DP72/CKX41; Olympus). In normal cells, JC-1 remained as a red fluorescent monomer, whereas during the induction of apoptosis the mitochondrial potential collapsed and hence JC-1 formed aggregates producing green fluorescence.

### 4.8. Measurement of Reactive Oxygen Species (ROS)

The intracellular oxidative bursts were evaluated using the DCFH-DA method. Cells were treated with 10 μM of DCFH-DA for 0.5 h at 37 °C under 5% CO_2_. Afterwards, intracellular oxidative burst images were measured using an inverted fluorescence microscope (DP72/CKX41; Olympus). In addition, the amount of H_2_O_2_ was measured using the Amplex Red Hydrogen Peroxide/Peroxidase assay kit (Life Technologies, Carlsbad, CA, USA) as described by the manufacturer. In the presence of peroxidase, the Amplex Red reagent reacted with H_2_O_2_ to produce a red-fluorescent oxidation product. Briefly, 2.5 × 10^4^ cells/well were treated with indicated compounds for 24 h in 48-well culture plates. After that, the cells were washed twice with PBS and then 200 μL of the Amplex Red reaction mixture was added into each microplate well. The mixture was incubated at 37 °C for 15 min, and the detection of H_2_O_2_ in each well was recorded as an increase in Amplex Red fluorescence using a fluorescence microplate reader (SpectraMax M5; Molecular Devices, Sunnyvale, CA, USA). Amplex Red fluorescence was measured at excitation and emission wavelengths of 560 and 590 nm, respectively. Fluorescence intensity was normalized by comparing the data as a percentage relative to the control group.

### 4.9. Real Time Quantitative PCR Analysis of mRNA Expression

Real time quantitative qPCR, using an ABI 7300 Sequence Detection System (Applied Biosystems, Foster City, CA, USA), was performed for quantification of mRNA. PCR amplifications of target mRNA genes were carried out in conjunction with Power SYBR Green PCR Master Mix (Applied Biosystems) according to the manufacturer’s instructions. Each cDNA sample was tested in triplicate. The following temperature parameters were 95 °C/10 min, 40 cycles of 95 °C/15 s, 60 °C/1 min and dissociation stage was 95 °C/15 s, 60 °C/15 s and 95 °C/15 s. The following primer pairs were used: forward 5'-CGGATGAAGAGAGGCATGTT-3' and reverse 5'-TTGTTTCTCATGGACCACCA-3' for SOD1, forward 5'-TCATGCATGCAAATCCTTGT-3' and reverse 5'-GGCCACCTGTAACTTCTCCA-3' for SOD2, forward 5'-AGATGCTGCATGGTCGTCTGTTGTTCT-3' and reverse 5'-TCCATCCCGCTGGAAGTTCTCAAT-3' for catalase, and forward 5'-TGGTATCGTGGAAGGACTCATGAC-3' and reverse 5'-ATGCCAGTGAGCTTCCCGTTCAGC-3' for GAPDH. Values of relative mRNA expression were obtained by using the software SDS version 1.2.3 (Sequence Detection Systems 1.2.3-7300 Real Time PCR System, Applied Biosystems) and the values were standardized by comparing with values from relative expression of GAPDH.

### 4.10. Statistical Analysis

Data were presented as means ± SEM. Statistical analysis of data was performed using analysis of variance (ANOVA), followed by Dunnett’s *post-hoc* test for multiple comparisons with SPSS statistical software (SPSS, Inc., Chicago, IL, USA). A probability value of <0.05 was taken to indicate statistical significance.
